# Evidence for the involvement of sphingosine-1-phosphate in the homing and engraftment of hematopoietic stem cells to bone marrow

**DOI:** 10.18632/oncotarget.4710

**Published:** 2015-07-17

**Authors:** Mateusz Adamiak, Sylwia Borkowska, Marcin Wysoczynski, Malwina Suszynska, Magda Kucia, Gregg Rokosh, Ahmed Abdel-Latif, Janina Ratajczak, Mariusz Z. Ratajczak

**Affiliations:** ^1^ Stem Cell Institute at James Graham Brown Cancer Center, University of Louisville, Louisville, KY, USA; ^2^ Institute of Molecular Cardiology, University of Louisville, Louisville, KY, USA; ^3^ Department of Regenerative Medicine, Medical University of Warsaw, Warsaw, Poland; ^4^ Division of Cardiovascular Medicine, Gill Heart Institute, University of Kentucky, Lexington, KY, USA

**Keywords:** Pathology Section, S1P, SDF-1, CXCR4, stem cell homing, hematopoietic stem cells

## Abstract

The α-chemokine stromal-derived factor 1 (SDF-1), which binds to the CXCR4 receptor, directs migration and homing of CXCR4^+^ hematopoietic stem/progenitor cells (HSPCs) to bone marrow (BM) stem cell niches. Nevertheless, it is also known that CXCR4^−/−^ fetal liver-derived hematopoietic stem cells engraft into BM and that blockade of CXCR4 by its antagonist AMD3100 does not prevent engraftment of HSPCs. Because of this finding of SDF-1-CXCR4-independent BM homing, the unique role of SDF-1 in HSPC homing has recently been challenged. While SDF-1 is the only chemokine that chemoattracts HSPCs, other chemoattractants for these cells have recently been described, including the bioactive phosphosphingolipid sphingosine-1-phosphate (S1P). To address the potential role of S1P in homing of HSPCs to BM, we performed hematopoietic transplants into mice deficient in BM-expressed sphingosine kinase 1 (Sphk1^−/−^) using hematopoietic cells from normal control mice as well as cells from mice in which floxed CXCR4 (CXCR4^fl/fl^) was conditionally deleted. We observed the presence of a homing and engraftment defect in HSPCs of Sphk1^−/−^ mice that was particularly profound after transplantation of CXCR4^−/−^ BM cells. Thus, our results indicate that BM-microenvironment-expressed S1P plays a role in homing of HSPCs. They also support the concept that, in addition to the SDF-1-CXCR4 axis, other chemotactic axes are also involved in homing and engraftment of HSPCs.

## INTRODUCTION

Stromal-derived factor 1 (SDF-1), which binds to the seven-transmembrane-spanning G_αI_ protein-coupled receptor CXCR4, is unique among the family of chemokines, because it chemoattracts hematopoietic stem/progenitor cells (HSPCs) [1–3]. Since the CXCR4 receptor is expressed on HSPCs, SDF-1 plays an important role in regulating the trafficking of these cells and facilitates their homing and engraftment in BM after transplantation. Furthermore, SDF-1 is involved in the subsequent retention of HSPCs in BM stem cell niches [3–6].

Nevertheless, the role of the SDF-1-CXCR4 axis in stem cell homing to BM has been recently challenged by several observations that support the involvement of SDF-1-CXCR4-independent homing mechanisms [7–9]. This evidence is based on observations that i) CXCR4^−/−^ fetal liver HSPCs home to BM in an SDF-1-independent manner [7], ii) homing of murine HSPCs made refractory to SDF-1 by incubation and co-injection with a CXCR4 receptor antagonist (AMD3100) is normal or only mildly reduced [8], and iii) HSPCs in which CXCR4 has been knocked down by means of an SDF-1 intrakine strategy are still able to engraft [9]. It is also known that iv) myeloablative conditioning for transplantation induces a highly proteolytic microenvironment in BM that leads to proteolytic degradation of SDF-1 and thus severely attenuates its chemotactic homing gradient [10].

Evidence has accumulated that one of the most potent chemotactic factors for HSPCs is the bioactive phosphosphingolipid sphingosine-1-phosphate (S1P) [11]. An S1P gradient in PB also plays a crucial role in mobilization of HSPCs from BM into PB [11–16]. The level of S1P in BM is regulated by a balance in activity between type 1 SP-1 kinase (Sphk1) and S1P lyase, which degrades S1P. Interestingly, in the past we observed that mice in which S1P lyase is inhibited by deoxypyridoxin (DOP) display an impaired release of HSPCs from BM to PB in response to mobilizing agents [11, 16]. This suggested the involvement of S1P in retention of HSPCs in BM. Based on this finding, we hypothesized that S1P plays a role also in homing and engraftment of HSPCs to BM and is involved in SDF-1-independent homing [10–12] what has been subsequently supported by observation that Sphk1-KO mice engraft poorly with WT BM cells exposed to AMD3100 [16]. To address this issue more directly and to shed more light on the role of BM-expressed S1P in the homing process, we performed homing experiments in Sphk1-deficient mice, transplanting these animals with wild type (WT) or CXCR4^−/−^ HSPCs [17].

We report here a defect in homing as well as engraftment of WT and, in particular, CXCR4^−/−^HSPCs in Sphk1^−/−^ mice. Thus, our data indicate that BM-microenvironment-expressed S1P plays a role in the homing of HSPCs and supports the concept that other chemotactic axes in addition to the SDF-1-CXCR4 axis are involved in homing and engraftment of HSPCs.

## RESULTS

### Normal steady-state hematopoiesis in Sphk1^−/−^ mice

**Figure 1:**
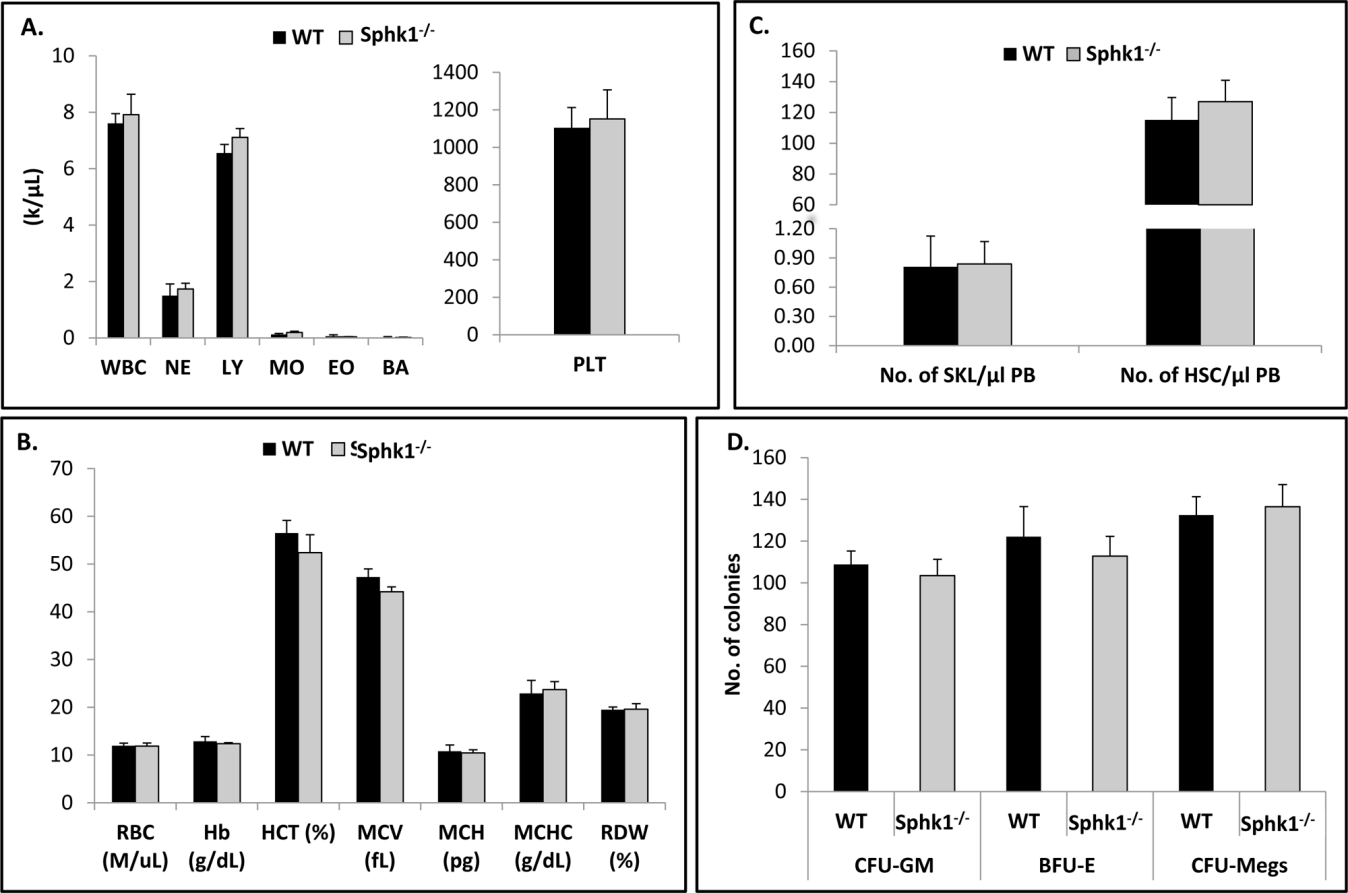
Sphk1 deficiency does not affect hematological homeostasis Bone marrows of WT and Sphk1^−/−^ mice were isolated and evaluated for peripheral blood white blood cell **A.** and red blood cell **B.** parameters as well as the number of SKL cells in BM **C.** and *in vitro* clonogenic progenitor cells **D.** The data represent an average of at least six mice per experimental group.

Since Sphk1 is the main BM-expressed isoform of Sphk, we became interested in the basic hematopoietic parameters of animals that lack expression of this enzyme. Figure 1A, 1B shows that Sphk1^−/−^ animals, in comparison with WT controls, display normal peripheral blood counts in all three major hematopoietic lineages. Moreover, these animals also have normal numbers of Sca-1^+^c-kit^+^Lin^−^ (SKL) cells enriched for HSPCs in BM (Figure 1C). Furthermore, by employing *in vitro* clonogenic assays, we found that BM cells isolated from Sphk1^−/−^ mice grow similar numbers of CFU-GM, BFU-E, and CFU-Meg colonies as normal control mice (Figure 1D).

### Defective homing of WT BM cells in Sphk1^−/−^ mice

**Figure 2:**
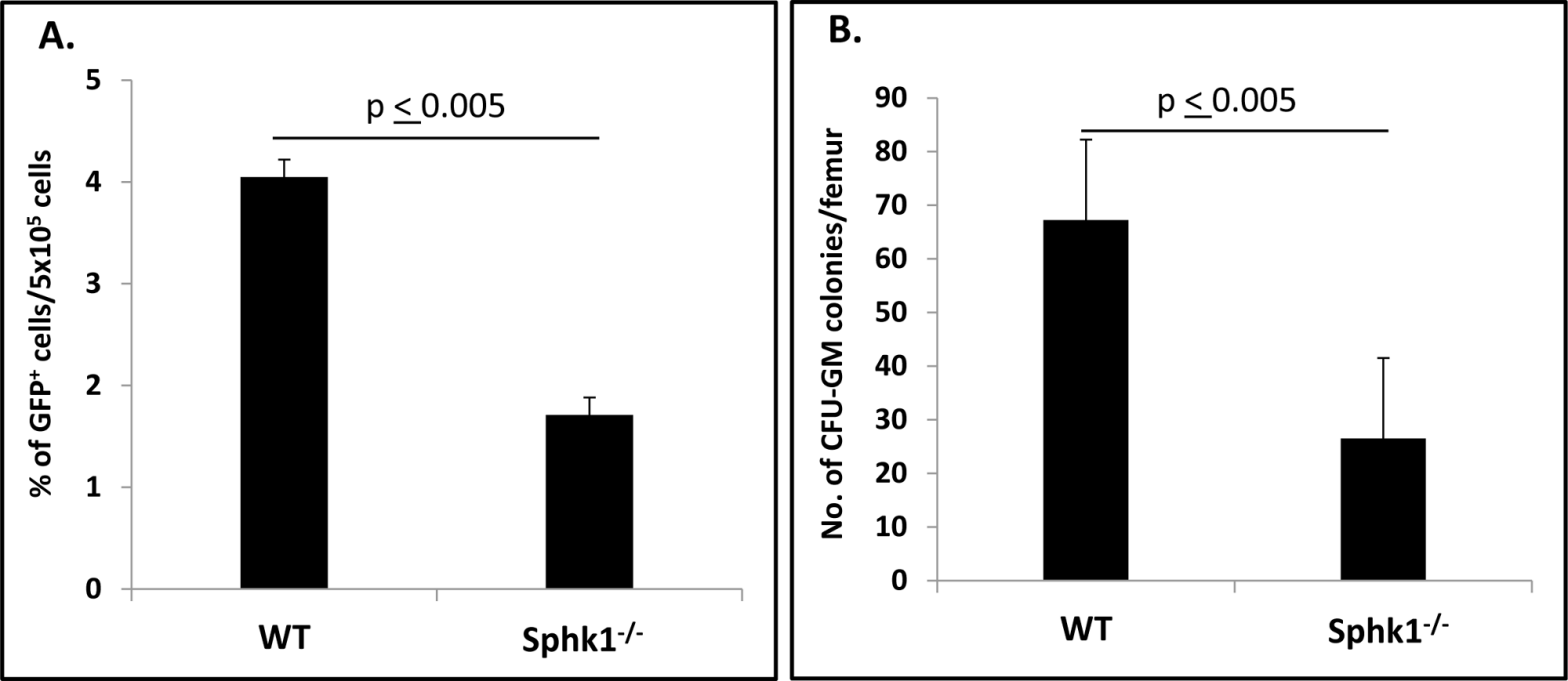
A defect in homing of HSPCs from GFP^+^ mice in Sphk1^−/−^ BM **Panel A.** Lethally irradiated Sphk1^−/−^ mice (six mice per group) were transplanted with 5 × 10^6^ GFP^+^ bone marrow mononuclear cells (BMMNCs); 24 hours after transplantation femoral BMMNCs were harvested; the number of GFP^+^ cells in murine BM was evaluated by FACS (panel A); and the number of clonogenic GFP^+^ CFU-GM progenitors was enumerated in an *in vitro* colony assay (panel B). No colonies were formed in lethally irradiated and non-transplanted mice (irradiation control). The data in panels A and B represent the combined results from two independent experiments (*n* = 10). *p < 0.005.

Homing or lodging of HSPCs to BM after transplantation precedes their engraftment and expansion in the hematopoietic microenvironment [3–6]. To study the homing process of HSPCs in Sphk1^−/−^ animals, we transplanted BM cells isolated from green fluorescent protein (GFP) mice into lethally irradiated normal control or Sphk1^−/−^ animals. Twenty-four hours after transplantation, the mice were sacrificed, and we evaluated the number of GFP^+^ cells in BM by FACS (Figure 2A) and the number of clonogenic progenitors that lodged during this time to BM, and we were able to grow *in vitro* GFP^+^ colonies after isolation from the long bones (Figure 2B). We found that Sphk1^−/−^ mice had significantly lower numbers of GFP^+^ cells and GFP^+^ clonogeneic progenitors in BM compared with normal control animals.

### A defect in short-and long-term BM engraftment of HSPCs in Sphk1^−/−^ animals

The outcome of a hematopoietic transplant is a function of homing/lodging efficiency and the number of transplanted cells. To study the role of BM-expressed S1P in the homing of HSPCs, we transplanted into Sphk1^−/−^mice normal control BM cells as well as CXCR4^fl/fl^ BM cells in which expression of CXCR4 had been efficiently deleted by Cre-recombinase [17]. Our preliminary results indicated that the chemotactic responsiveness of normal control and CXCR4^−/−^ BM clonogenic progenitors to S1P gradients was similar (Supplementary Figure S1).

**Figure 3:**
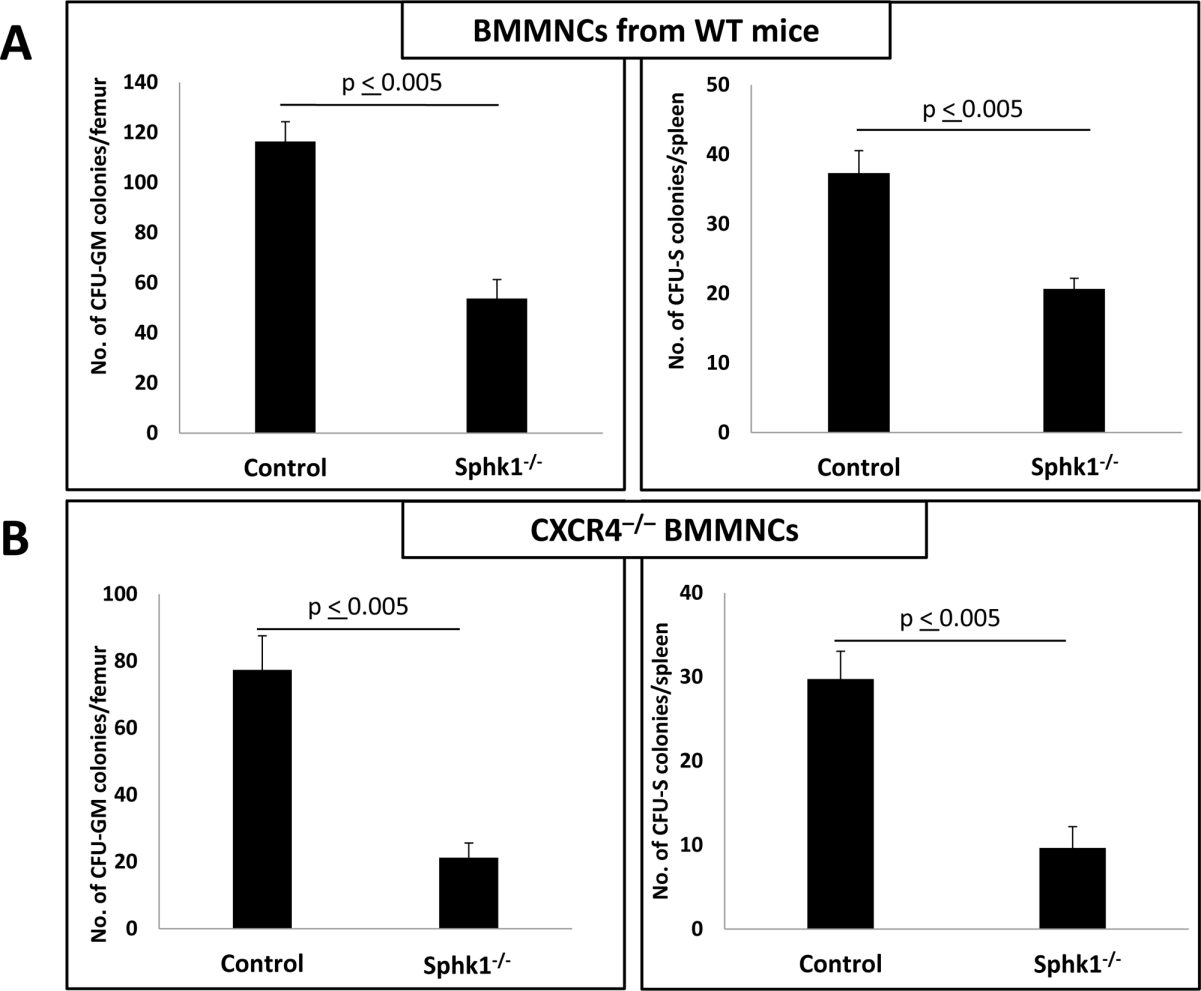
A defect in short-term engraftment of WT and CXCR4^−/−^ HSPCs in Sphk1^−/−^ BM **Panel A.** Lethally irradiated mice (six mice per group) were transplanted with 1.5 × 10^5^ bone marrow mononuclear cells (BMMNCs) from WT mice, and 11 days after transplantation spleens were removed and femoral BMMNCs were harvested for counting the number of CFU-S colonies and plating to count the number of CFU-GM colonies, respectively. **Panel B.** In similar experiments, mice were lethally irradiated and transplanted with 1.5 × 10^5^ BMMNCs from CXCR4^−/−^ mice. Eleven days after transplantation, spleens were removed and femoral BMMNCs were harvested for counting the number of CFU-S colonies and plating to count the number of CFU-GM colonies, respectively. The data in panels A and B represent the combined results from three independent experiments (*n* = 9). *p < 0.005.

Thus, we transplanted normal BM cells into normal (control) mice or Sphk1^−/−^ animals and evaluated the number of day-11 CFU-GM colonies present in BM as well as the number of day-11 spleen colonies formed by CFU-S (Figure 3A) and found impaired homing of HSPCs to BM in Sphk1^−/−^ mice. Similar and even more profound defects in the number of day-11 CFU-GM colonies and day-11 CFU-S colonies were observed after transplantation of CXCR4^−/−^ BM cells (Figure 3B).

**Figure 4:**
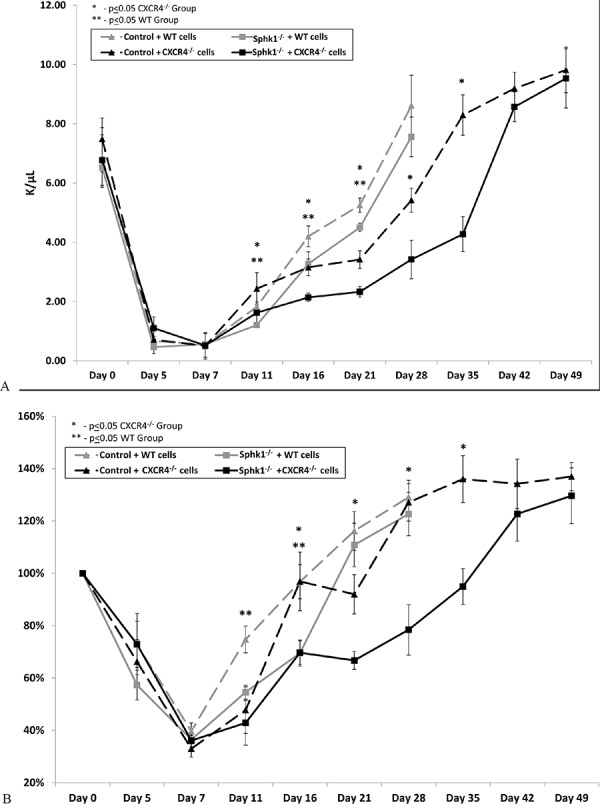
A defect in long-term engraftment of WT and CXCR4^−/−^ HSPCs in Sphk1^−/−^ BM Lethally irradiated Sphk1^−/−^ mice were transplanted with 1 × 10^6^ BMMNCs from WT or CXCR4^−/−^ cells. White blood cells (**panel A**) and platelets (**panel B**) were counted at intervals (at 0, 5, 7, 11, 16, and 21 days and, for CXCR4^−/−^ transplant, additionally at 28, 35, 42, and 49 days after transplantation). For platelets count changes have been shown in % in comparison to day 0 for which platelet counts were assumed to be 100%. Results are combined from two independent experiments (six mice per group, *n* = 12). *p < 0.05.

Based on these results, we transplanted normal BM cells or CXCR4^−/−^ BM cells into lethally irradiated normal or Sphk1^−/−^ mice and followed the recovery of white blood cells and platelet counts in these animals (Figure 4A, 4B). We found that Sphk1^−/−^animals, compared with WT mice, showed a defect in recovery of leucocytes and platelets and that this defect was more pronounced if mice were transplanted with CXCR4^−/−^ BM cells. As shown in Figure 4, the recovery of peripheral blood counts in Sphk1^−/−^ mice after transplantation of CXCR4^−/−^ cells was delayed by two weeks compared with normal mouse recipients.

## DISCUSSION

The salient observation of this study is that BM-expressed S1P is involved in homing and engraftment of HSPCs to BM. Despite the fact that the SDF-1-CXCR4 axis is crucial for retention of HSPCs in BM niches and that Sphk1^−/−^ mice do not show any hematopoietic defects under steady-state conditions, our results support the involvement of S1P in this process and the existence of an SDF-1-independent, S1P-mediated homing mechanism.

Overall, the developmental and postnatal migration of HSPCs is still not well understood. HSPCs migrate during embryonal development, colonizing different organs where hematopoiesis is initiated. In the second trimester of gestation, they colonize fetal liver, which is a major hematopoietic organ at this stage of development [18–20]. Despite the fact that SDF-1 is considered to be the most important factor regulating migration of HSPCs, surprisingly, murine embryos with CXCR4 or SDF-1 knocked out have a normal number of myeloid HSPCs in fetal liver [21–24]. Because fetal liver is colonized by HSPCs migrating from the aorta-gonad-mesonephros (AGM) region, this result suggests that this process is mediated by chemoattractants other than SDF-1 [21–24]. Moreover, as mentioned in the introduction, HSPCs isolated from CXCR4^−/−^ murine fetal liver home to BM in an SDF-1-independent manner [7], and it is also well known that homing of murine HSPCs made refractory to SDF-1 by incubation and co-injection with a CXCR4 receptor antagonist (AMD3100) is normal or only mildly reduced [8]. Finally, as we demonstrate here, CXCR4^−/−^ BM cells generated by a Cre-recombinase strategy also home to BM after transplantation, although the timing of the homing and engraftment process is delayed.

All this supports the conclusion that, in addition to the SDF-1-CXCR4 axis, additional redundant mechanisms exist. Taking all this into consideration, we focused our investigation on the potential role of S1P. This phosphosphingolipid is a potent chemoattractant for HSPCs, even more potent than SDF-1 if employed at the physiological doses that are encountered in biological fluids [11]. Because of its chemical structure, S1P, in contrast to peptide-based SDF-1, is resistant, to destruction by proteolytic enzymes, which are highly upregulated in the BM microenvironment when it is irradiated or conditioned by chemotherapy [10]. S1P has been demonstrated to be involved in trafficking of HSPCs, and, as recently demonstrated by us and others, S1P present in PB is involved in egress of HSPCs from BM during the mobilization process [11–16].

Interestingly, our previous research demonstrated that mobilization of HSPCs is impaired in mice after inhibition of S1P lyase, which degrades S1P and thus increases its level in BM [25]. This observation, along with the fact that S1P is upregulated after conditioning for transplantation in the BM microenvironment [11], prompted us to study its role in the homing process.

We report here that Sphk1^−/−^ mice, deficient in S1P level in BM, display impaired homing of transplanted HSPCs. Since these mice have a normal level of SDF-1 in BM, the lack of S1P in the hematopoietic microenvironment was compensated after transplantation of normal control BM cells by chemoattraction through the SDF-1-CXCR4 axis. However, if we performed transplants into Sphk1^−/−^ mice employing CXCR4^−/−^ BM cells, we observed delayed long-term engraftment of the transplanted cells, and this experiment revealed the importance of the S1P homing mechanism.

However, the fact that CXCR4^−/−^ cells were finally able to engraft in Sphk1^−/−^ mice suggests the existence of other chemoattractants that are involved in this process in addition to SDF-1 and S1P. In support of this inference, it has been demonstrated that another bioactive phosposphingolipid, ceramide-1-phosphate (C1P), is also a potent chemoattractant for HSPCs [10]. We have reported that, like the S1P level, the C1P level is also increased in BM after myeloablative conditioning for transplantation [10, 11]. The problem with studying the possible involvement of C1P in the homing process is that the receptor/s for C1P has not yet been identified, and thus the appropriate murine knockout experimental models are not available.

Other potential factors involved in the homing process are extracellular nucleotides such as ATP or UTP [26–28]. We have recently reported that the concentration of extracellular nucleotides also increases in BM after irradiation [29, 30]. Thus, it would be interesting to see the effect of extracellular nucleotide receptor blockage on homing and engraftment of HSPCs, and we are currently planning such experiments.

Despite the fact that some crucial chemotactic factors for HSPCs, including SDF-1, S1P, C1P, and extracellular nucleotides (e.g., ATP and UTP), have been identified, we cannot exclude the possibility that there are other molecules and mechanisms involved in homing that could compensate for an SDF-1-CXCR4 axis deficiency. For example, the involvement of Ca2^+^-sensing mechanisms in the migration of HSPCs has been described [31]. A similar H^+^-sensing mechanism could also be involved, but this requires further study.

It is well known that SDF-1 can also bind to the CXCR7 receptor, but the involvement of the SDF-1-CXCR7 axis in the homing of HSPCs is based on inconclusive evidence. Nevertheless, it would be interesting to perform homing experiments in Sphk1^−/−^ animals with HSPCs in which both CXCR4 and CXCR7 receptors are eliminated from the cell surface. An alternative strategy would be to perform transplant experiments into Sphk1^−/−^ mice in which stromal SDF-1 is conditionally knocked out.

An important question is how conditional CXCR4 knockout BM cells engrafted in our mouse models could be theoretically explained by an SDF-1 priming phenomenon [32]. It is known that several soluble factors released during conditioning for transplantation, including elements of innate immunity, such as cleavage fragments of C3 [33]; cationic antimicrobial peptides, such as cathelicidin (LL-37) [34] and β2-defensin [35]; and prostaglandin E2 (PGE2) [36], a member of the eicosanoid family, significantly enhance responsiveness of the SDF-1-CXCR4 axis. However, since CXCR4 was deleted by ~99% by Cre-recombinase in the HSPCs employed in our experiments, SDF-1 priming effects are most likely not involved.

In these experiments we did not address which of the S1P receptors is most crucial for the observed phenomena, but, based on the responsiveness of HSPCs to an S1P gradient, the most likely candidate in S1P-mediated homing is the S1P type 1 receptor (S1P_1_R) [11–16].

**Figure 5:**
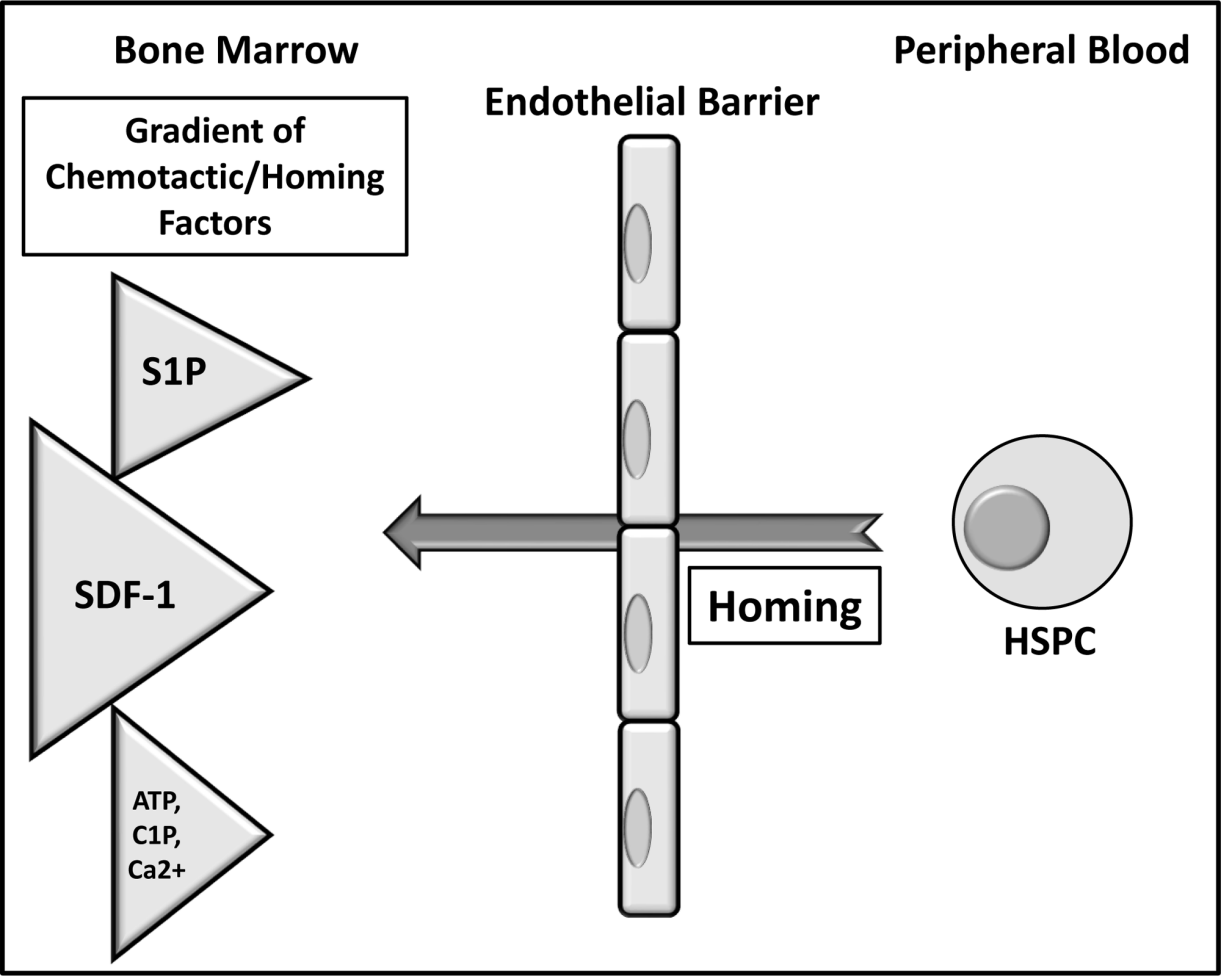
The proposed major mechanism underlying the processes of homing of HSPCs HSPCs infused into blood respond to a gradient of SDF-1 between bone marrow and blood; however, conditioning for transplantation by chemoradiotherapy may also upregulate other homing factors, such as S1P and C1P, and increase the levels of extracellular nucleotides (ATP and UTP). Homing is also regulated by Ca^2+^ gradient in the BM microenvironment. Most likely, there are also other unidentified yet factors involved in the homing process.

In conclusion, we demonstrated a defect in homing as well as engraftment of WT HSPCs and, most importantly, CXCR4^−/−^ HSPCs in Sphk1^−/−^ mice. Thus, our results indicate that S1P expressed in the BM-microenvironment plays a role in homing of HSPCs and supports the concept that other chemotactic axes are involved in homing and engraftment of HSPCs in addition to the SDF-1-CXCR4 axis (Figure 5). Further studies are needed to evaluate the involvement of other potential homing factors, such as C1P, extracellular nucleotides (e.g., ATP), Ca^2+^ sensing mechanism, PGE2 involvement and a role of hyaluronic acid - CD44 interaction [11, 12, 27, 31, 36, 39–42], that may compensate for SDF-1-and S1P-mediated homing.

## MATERIAL AND METHODS

### Animals

Sphk1^−/−^ 6–8-week-old mice and age-and sex-matched littermates, all employed in homing experiments, as well as green immunofluorescence protein (GFP)-expressing mice were purchased from The Jackson Laboratory, Bar Harbor, ME. Cxcr4^fl/fl^ mice (provided by Dr. Yong-Rui Zou, The Feinstein Institute for Medical Research) were crossed with B6.Cg-Tg(UBC-cre/ERT2)1Ejb/J mice (Jackson Labolatory) to generate CXCR4^fl/fl^Cre^Tg/−^ mice [17]. To delete Cxcr4, mice were injected intraperitoneally with tamoxifen (80 mg/kg/day, Sigma-Aldrich) for five consecutive days. The efficiency of recombination was evaluated in peripheral blood and bone marrow after the last tamoxifen injection and was greater than 99%. Animal studies were approved by the Animal Care and Use Committee of the University of Louisville (Louisville, KY).

### Peripheral blood (PB) parameter counts

To obtain leukocyte and red blood cells counts, 50 microliters of PB were taken from the retro-orbital plexus of the mice into EDTA-coated Microvette tubes (Sarstedt Inc., Newton, NC, USA) and run within 2 h of collection on a HemaVet 950FS hematology analyzer (Drew Scientific Inc., Oxford, CT, USA) as described [11].

### FACS analysis

The following monoclonal antibodies were employed to perform analytical staining of Lin^−^/Sca-1^+^/c-kit^+^ (SKL cells) and Lin^−^/Sca-1^+^/CD45^+^ (HSCs): fluorescein isothiocyanate (FITC) anti-CD117 (c-kit) (clone2B8; BioLegend, San Diego, CA) and phycoerythrin (PE)-Cy5 anti-mouse Ly-6A/E (Sca-1) (clone D7; eBioscience™, San Diego, CA). All anti-mouse lineage markers (Lin) were conjugated with PE and purchased from BD Biosciences: anti-CD45R/B220 (clone RA3-6B2); anti-Ter-119 (clone TER-119); anti-CD11b (clone M1/70); anti-T-cell receptor β (TCRβ; clone H57-597); anti-Gr-1 (clone RB6-8C5); anti-TCRγδ (clone GL3); and anti-CD45 (clone 30-F11). All monoclonal antibodies were added at saturating concentrations, and the cells were then incubated for 30 min on ice, washed twice, resuspended in RPMI-1640 plus 2% fetal bovine serum, and analyzed with an LSR II flow cytometer (BD, San Diego, CA, USA) [11].

### Clonogenic *in vitro* assay

After red blood cells (RBCs) from PB were lysed (Pharm Lyse buffer, BD Biosciences, San Jose, CA), the nucleated cells were subsequently washed twice and used for CFU-GM colony formation, and cells were counted and resuspended in human methylcellulose base media provided by the manufacturer (R&D Systems, Inc., Minneapolis, MN), supplemented with 25 ng/ml recombinant murine granulocyte macrophage colony-stimulating factor (mGM-CSF) and 10 ng/ml recombinant murine interleukin 3 (mIL-3; Millipore, Billerica, MA). Cultures were incubated for 7 days and then scored for the number of CFU-GM colonies under an inverted microscope. We cultured 1 × 10^6^ peripheral blood mononuclear cells (PBMNCs)/dish, and final results were recalculated based on the number of PBMNCs per 1 μl of PB. Each clonogenic test was performed in duplicate. To evaluate the number of clonogenic progenitor cells, bone marrow mononuclear cells (BMMNCs) were supplemented with erythropoietin (EPO, 5 unit/ml, Stem Cell Tech) plus stem cell factor (SCF, 5 ng/ml), resuspended in methylcellulose base medium for burst-forming units (BFU-E), supplemented with thrombopoietin (TPO, 100 ng/ml) plus interleukin-3 (mIL-3, 10 ng/ml), and resuspended in plasma cloth cultures for determining the number of CFU-megakaryocytes (Megs) and CFU-GM colonies, performed as above. Cultures were incubated for 7 days (37°C, 95% humidity, and 5% CO_2_), at which time they were scored under an inverted microscope for the number of colonies, as described [11].

### Short-term homing experiments

Mice were irradiated with a lethal dose of γ-irradiation (1000 cGy). After 24 h, the animals were transplanted (by tail vein injection) with 5 × 10^6^ BM cells from GFP mice. At 24 h after transplant, BM cells from the femurs were isolated via Ficoll-Paque and divided. Some cells (5 × 10^5^) were incubated with Vybrant^®^ DyeCycle™ Ruby stain (Invitrogen, CA) and analyzed on a flow cytometer. The rest of the cells were plated in serum-free methylcellulose cultures and stimulated to grow CFU-GM colonies with granulocyte macrophage-colony stimulating factor (GM-CSF, 25 ng/ml) and interleukin 3 (IL-3, 10 ng/ml). After 7 days of incubation (37°C, 95% humidity, and 5% CO_2_) the number of colonies was scored under an inverted microscope [37].

### Evaluation of engraftment

For engraftment experiments, mice were irradiated with a lethal dose of γ-irradiation (1000 cGy). After 24 h, mice were transplanted with 1.5 × 10^5^ BM cells from WT or Cxcr4^−/−^ mice by tail vein injection. Femora of transplanted mice were flushed with phosphate-buffered saline (PBS) on day 12 post-transplant. Purified via Ficoll-Paque, BM cells were plated in serum-free methylcellulose cultures and stimulated to grow CFU-GM colonies with G-CSF (25 ng/ml) plus IL-3 (10 ng/ml). After 7 days of incubation (37°C, 95% humidity, and 5% CO_2_) the number of colonies was scored under an inverted microscope. Spleens were also removed, fixed in Telesyniczky's solution for CFU-S assays, and colonies were counted on the surface of the spleen [38].

### Recovery of leukocytes and platelets

For transplantation experiments, mice were irradiated with a lethal dose of γ-irradiation (1000 cGy). After 24 h, the mice were transplanted by tail vein injection with 1 × 10^6^ BM cells. Transplanted mice were bled at various intervals from the retro-orbital plexus to obtain samples for white blood cell and platelet counts as described [37].

### Chemotaxis assay

BMMNCs from WT and Cxcr4^−/−^ mice at a density of 1 × 10^6^ in 100 μl were aliqoted into 5-μm polycarbonate membrane inserts in a Costar Transwell 24-well plate (Costar Corning, Cambridge, MA, USA) for 3 h chemotaxis at 37°C. Medium with 0.5% BSA (650 μl/well) containing either no chemoattractant (negative control) or sphingosine-1-phosphate (0.1 μM, Cayman Chemical, Michigan, USA) was added to the lower chambers of the plate. Subsequently, cells in the lower chambers were harvested and assayed for the number of CFU-GM colony-forming units. Briefly, cells were resuspended in human methylcellulose base medium provided by the manufacturer (R&D Systems), supplemented with GM-CSF (25 ng/ml) and IL-3 (10 ng/ml) for determining the number of CFU-GM colonies. Cultures were incubated for 7 days, at which time they were scored under an inverted microscope for the number of colonies [11].

### Statistical analysis

All results are presented as mean ± SD. Statistical analysis of the data was done using Student's *t*-test for unpaired samples, with *p* ≤ 0.05 considered significant.

## SUPPLEMENTARY FIGURE


