# Supplementary material for: Evidence for the involvement of sphingosine-1-phosphate in the homing and engraftment of hematopoietic stem cells to bone marrow
Source: Oncotarget. 2015 Jul 17;6(22):18819–28. doi: 10.18632/oncotarget.4710 (PMC4662458; doi:10.18632/oncotarget.4710)
Supplement: Supplementary file 1 [file oncotarget-06-18819-s001.pdf]

## SUPPLEMENTARY FIGURE

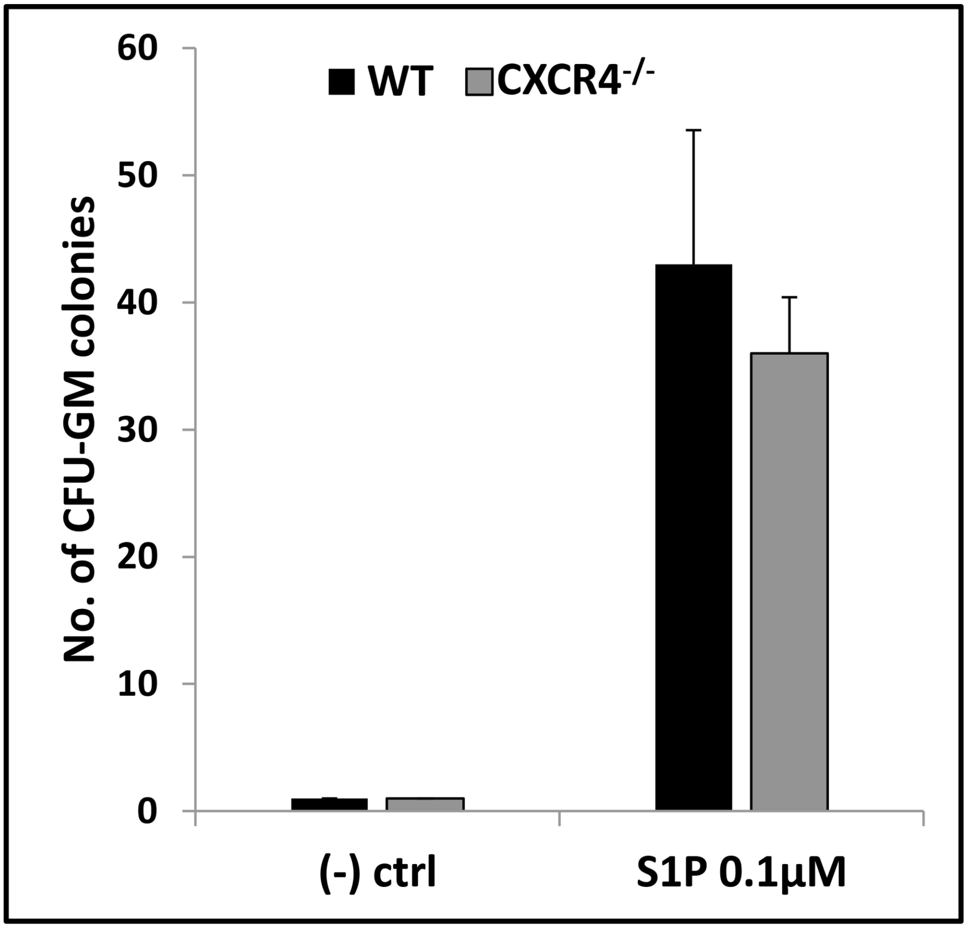


**Supplementary Figure S1: The chemotactic responsiveness of BM-derived CFU-GM clonogenic progenitors isolated from WT and CXCR4$^{-/-}$ mice to an S1P gradient.** Results are combined from three independent experiments.
